# Listening Difficulties in Children: Behavior and Brain Activation Produced by Dichotic Listening of CV Syllables

**DOI:** 10.3389/fpsyg.2020.00675

**Published:** 2020-04-16

**Authors:** David R. Moore, Kenneth Hugdahl, Hannah J. Stewart, Jennifer Vannest, Audrey J. Perdew, Nicholette T. Sloat, Erin K. Cash, Lisa L. Hunter

**Affiliations:** ^1^Communication Sciences Research Center, Cincinnati Children’s Hospital Medical Center, Cincinnati, OH, United States; ^2^Department of Otolaryngology, University of Cincinnati College of Medicine, Cincinnati, OH, United States; ^3^Manchester Centre for Audiology and Deafness, University of Manchester, Manchester, United Kingdom; ^4^Department of Biological and Medical Psychology, Faculty of Psychology, University of Bergen, Bergen, Norway; ^5^Department of Psychiatry, Haukeland University Hospital, Bergen, Norway; ^6^Department of Radiology, Haukeland University Hospital, Bergen, Norway; ^7^Division of Psychology and Language Sciences, University College London, London, United Kingdom; ^8^Division of Neurology and Pediatric Neuroimaging Research Consortium, Cincinnati Children’s Hospital Medical Center, Cincinnati, OH, United States; ^9^Department of Pediatrics, University of Cincinnati College of Medicine, Cincinnati, OH, United States

**Keywords:** auditory processing disorder, hearing loss, ECLiPS, laterality index, LiSN-S, NIH Cognition Toolbox, speech evoked fMRI, interaural level difference

## Abstract

Listening difficulties (LiD) are common in children with and without hearing loss. Impaired interactions between the two ears have been proposed as an important component of LiD when there is no hearing loss, also known as auditory processing disorder (APD). We examined the ability of 6–13 year old (y.o.) children with normal audiometric thresholds to identify and selectively attend to dichotically presented CV syllables using the Bergen Dichotic Listening Test (BDLT; www.dichoticlistening.com). Children were recruited as typically developing (TD; *n* = 39) or having LiD (*n* = 35) based primarily on composite score of the ECLiPS caregiver report. Different single syllables (ba, da, ga, pa, ta, ka) were presented simultaneously to each ear (6 × 36 trials). Children reported the syllable heard most clearly (non-forced, NF) or the syllable presented to the right [forced right (FR)] or left [forced left (FL)] ear. Interaural level differences (ILDs) manipulated bottom-up perceptual salience. Dichotic listening (DL) data [correct responses, laterality index (LI)] were analyzed initially by group (LiD, TD), age, report method (NF, FR, FL), and ILD (0, ± 15 dB) and compared with speech-in-noise thresholds (LiSN-S) and cognitive performance (NIH Toolbox). fMRI measured brain activation produced by a receptive speech task that segregated speech, phonetic, and intelligibility components. Some activated areas [planum temporale (PT), inferior frontal gyrus (IFG), and orbitofrontal cortex (OFC)] were correlated with dichotic results in TD children only. Neither group, age, nor report method affected the LI of right/left recall. However, a significant interaction was found between ear, group, and ILD. Laterality indices were small and tended to increase with age, as previously reported. Children with LiD had significantly larger mean LIs than TD children for stimuli with ILDs, especially those favoring the left ear. Neural activity associated with Speech, Phonetic, and Intelligibility sentence cues did not differ significantly between groups. Significant correlations between brain activity level and BDLT were found in several frontal and temporal locations for the TD but not for the LiD group. Overall, the children with LiD had only subtle differences from TD children in the BDLT, and correspondingly minor changes in brain activation.

## Introduction

Listening is often considered to be the active counterpart of passive hearing; “paying thoughtful attention to sound” (Keith et al., 2019; after Merriam-Webster). By definition, therefore, children with LiD may have problems with thought, attention, or hearing. In practice, a considerable number of children seen at audiology clinics who have LiD are, on further testing, found to have normal audiograms, the pure-tone detection, gold-standard measure of hearing (Hind et al., 2011). For these children, a wide variety of symptoms are reported by caregivers (American Academy of Audiology, 2010; Moore and Hunter, 2013) that may be summarized as difficulty responding to meaningful sounds while ignoring irrelevant sounds. For at least 40 years, children with these symptoms have, following further testing, been diagnosed by some audiologists as having an auditory processing disorder (APD), but that diagnosis has not gained universal acceptance (Moore, 2018), so we will generally refer to the symptoms here by the more generic and non-diagnostic term LiD.

Impaired interactions between the two ears have been proposed as an important component of LiD, based mainly on studies of DL, the simultaneous presentation of different acoustic signals to the two ears (Broadbent, 1956; Kimura, 1961; Keith, 2009). However, other aspects of binaural interaction, including binaural (Moore et al., 1991; Pillsbury et al., 1991) and spatial (Cameron and Dillon, 2007) release from masking have received substantial attention as contributors to LiD in adults and in children. Many other aspects of hearing and listening have also been studied in children with LiD (Moore et al., 2010; Weihing et al., 2015; de Wit et al., 2016; Wilson, 2018) leading, overall, to the emergence of two dominant hypotheses concerning the nature of the problem experienced by these children. The first, and more traditional hypothesis is that a disorder, (C)APD, is primarily a result of impaired processing of auditory neural signals in the central auditory system, defined as the brain pathway from the auditory nerve to the auditory cortex (Rees and Palmer, 2010). The second, disruptive hypothesis is that LiD are due primarily to impaired speech/language synthesis, inattention, or other executive function impairment in cortical processing of auditory information beyond the central auditory system. The study reported here was motivated by an attempt to distinguish between these hypotheses.

There is a rich history of studies of DL in children going back at least to Kimura (1963). Many of the early studies included children with a variety of learning problems, of which reading disability was perhaps the most common. Interestingly, several of these studies appeared to equate language and other abilities now considered to be cognitive with central auditory processing. However, Roeser et al. (1983), studying dichotic CV syllables in both TD 6–10 y.o. children and children with language impairment, concluded that the “dichotic CV syllables test has limited prognostic value in identifying auditory processing dysfunction in children classified in having a learning disability.”

More recently, some clinical DL tests have focused on listener reports of words (Keith, 2009), especially the spoken digits 1–10 (Musiek, 1983), that carry a substantial memory and executive control load in addition to their linguistic and acoustic demands. Nevertheless, dichotic digits, often described confusingly as a test of binaural integration (American Academy of Audiology, 2010; Brenneman et al., 2017), has become one of the most common clinical tests of APD (Emanuel et al., 2011). Various dichotic digit-based training programs have been proposed as interventions for the remediation of APD (Moncrieff et al., 2017). However, recent research (Cameron et al., 2016; Cameron and Dillon, 2020) has questioned whether dichotic digits testing involves any binaural interaction. These researchers found that performance on a *diotic* version of the test (presenting the same digits simultaneously to the two ears) correlates highly (*r* = 0.8) with performance on the dichotic version. The results suggest that, while binaural hearing may be disrupted during listening to dichotic digits, multiple, diverse abilities (acoustic discrimination, semantic identification, attentive listening, separation of two simultaneously presented sounds, accurate recall of heard digits) determine performance on these tasks.

As part of a larger Cincinnati Children’s Hospital (CCH) program to investigate the nature of LiD in 6–13 y.o. children with normal audiometric thresholds (SICLID), we examined those children’s ability to identify dichotically presented CV syllables using the BDLT (see www.dichoticlistening.com). In the BDLT (Hugdahl et al., 2009), two different CV syllables (from ba, da, ga, ka, pa, ta) are presented simultaneously, one to each ear, and the listener is asked either to report the first or most clearly heard syllable (NF condition), or selectively to report only that syllable presented to the left (FL) or to the right (FR) ear. In the NF condition, also known as the “Listen” mode, the proportion of syllables presented to the right ear that is correctly reported consistently exceeds the proportion presented to the left ear that is correctly reported, a REA.

The REA is a long established, robust, bottom-up, stimulus-driven, perceptual effect. Historically, it has been found to decrease with age (Kimura, 1963), and also to decrease in children with learning problems (Harris et al., 1983). Based on observations of adult patients with large temporal lobe lesions, and volunteers with sodium amytal silencing of a whole hemisphere, the REA was proposed to reflect left hemisphere dominance for processing of speech (Kimura, 1961). More recently, the REA has been reflected in left-hemifield dominant activation of the auditory cortex in studies using fMRI (Hugdahl et al., 1999; Rimol et al., 2005; Westerhausen and Hugdahl, 2010). It is modulated by top-down, cognitive influences (attention, executive function, working memory, training), reflected in FR and, particularly, FL performance (Kompus et al., 2012; Hugdahl and Westerhausen, 2016). An acoustic ILD between the syllables can offset the REA and thus serve as a physical measure (in dB) of a cognitive construct (Hugdahl et al., 2008; Westerhausen et al., 2009). For these reasons, as well as its simplicity and the extensive literature on it, the BDLT is well suited to investigate neural processes of listening in children. This study represents the first, to our knowledge, where BDLT has been used to examine children with LiD/APD.

Data from two other SICLID test suites, the LiSN-S listening of sentences in spatialized noise (Cameron and Dillon, 2007), and the NIH Cognition Toolbox (Weintraub et al., 2013), are briefly presented here to examine possible correlations with functions revealed by BDLT testing. In particular, we were interested to know how BDLT data related to specific measures of space- and talker-based grouping of sounds, and of presumed underpinning cognitive function.

Previous studies have shown that BDLT “Concentrate” modes (FL, FR) activate different brain regions in adults when contrasted with the Listen mode (Westerhausen and Hugdahl, 2010). Thus, FR activates a “dorsal attention network,” consisting of the rDLPFC and, weakly, lDLPFC and the bilateral occipital cortex. FL activates a “cognitive control network,” consisting of the bilateral angular gyrus, DLPFC, and anterior cingulate cortex (Westerhausen et al., 2010). We have taken another approach to examine the neural mechanisms underlying performance on the BDLT. Specifically, we used a sentence listening and speaker identification test to produce BOLD activation inside a 3T MRI scanner. We contrasted aspects of the sentence listening task to isolate components of receptive speech (speech, phonetics, intelligibility). We examined the relationship between BOLD activation (Scott et al., 2000; Halai et al., 2015) for each group (TD, LiD) and children’s performance by BDLT listening mode (NF, FL, FR) and interaural acoustic bias (ILD).

Testing the hypothesis that children with LiD have problems with cortical language, attention, and executive function beyond the central auditory system, the predictions of this study were that (i) children with LiD will perform normally on BDLT in Listen mode but will have difficulty in Concentrate mode, based on their overall tendency to perform poorly on cognitive tasks despite normal hearing; and (ii) children with LiD will have atypical top-down brain activation contrasts (Intelligibility, Speech), but typical Phonetic contrasts associated with BDLT performance. To investigate these predictions, we examined BDLT performance in normally hearing 6–13 y.o. children and correlated that performance with other tests of speech perception, cognition, and speech-evoked fMRI.

## Materials and Methods

### Participants

Children with LiD were recruited initially from a medical record review study of over 1100 children assessed for APD at CCH (Moore et al., 2018). Caregivers of children diagnosed with APD (including those with a “Disorder” or a “Weakness”) who responded to invitation to participate were sent questionnaires including the ECLiPS, below, and a background questionnaire on relevant demographic, medical (otology and neurology), and educational (learning disorders) issues. Those who completed and returned the questionnaires were invited to bring their child into the lab for a study visit. Over time, recruitment expanded to include the use of CCH IRB approved materials, advertising, and messages via print, electronic, social, and digital media at hospital locations and in the local and regional area for participation of families with children who had a “LiD,” or were “without any known or diagnosed learning problem.” Following a positive response and a brief telephone interview to screen for listening status, families were sent the same questionnaire pack and were invited for a study visit as described below.

**FIGURE 1:**
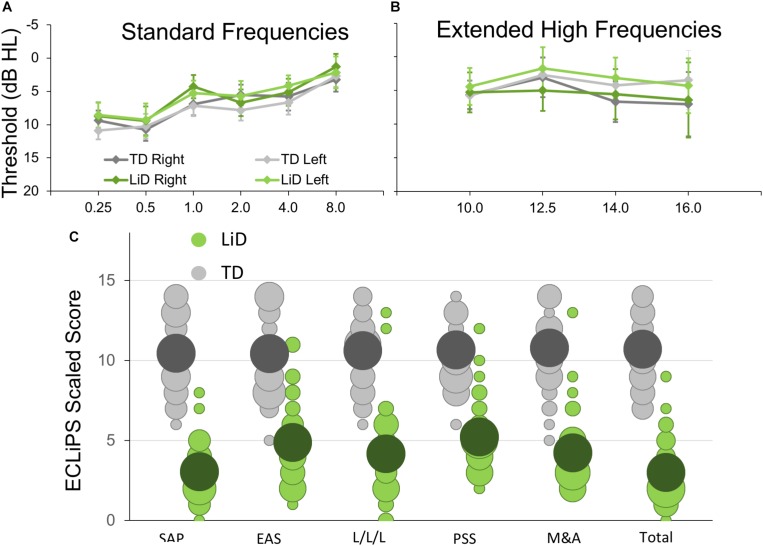
Children in study had no hearing loss but reduced caregiver evaluation of listening skills. **(A)** Mean (±95% CI) pure tone audiometric thresholds at standard frequencies (0.25–8.0 kHz) for children in each group. TD—typically developing, LiD—Listening difficulties. **(B)** Audiometric thresholds at extended high frequencies. **(C)** Caregiver evaluation using the ECLiPS (Barry and Moore, 2015; Barry et al., 2015). Scaled scores (normalized to mean = 10, SD = 3) shown separately for Total score, Speech and Auditory Processing (SAP), Environmental and Auditory Sensitivity (EAS), Language/Literacy/Laterality (L/L/L), Memory and Attention (M&A), and Pragmatic and Social Skills (PSS). Bubble size proportional to number of children achieving each scaled score.

Seventy four children aged 6–13 y.o. completed BDLT testing and most of the secondary behavioral testing. All of these children had normal hearing, bilaterally, defined as clear ears, A-type tympanometry, and pure tone thresholds ≤ 20 dB at octave frequencies between 0.25 and 8 kHz (Figure 1A) using standard audiometric procedures. Additional, extended high frequency audiometry (10–16 kHz; Figure 1B) was also obtained, but inclusion did not require any criterion level of performance at those frequencies. Seventy children received MRI scanning (95%).

The ECLiPS questionnaire (Barry and Moore, 2015; Roebuck and Barry, 2018) is a 38-item inventory asking users to agree or disagree (five-point Likert scale) with simple statements about their child’s listening and related skills. Total standardized ECLiPS scores ≥ 5 designated TD, and scores < 5, or a previous diagnosis of APD, designated LiD, resulting in 39 TD children (mean age 9.84 years, SD = 2.19) and 35 children with LiD (mean age 10.16, years; SD = 2.14; Figure 1C). Of the children in the LiD group previously diagnosed with APD (*n* = 9; see below), two scored 5 or more on the ECLiPS. These children were nevertheless included in the LiD group.

Demographics, audiological status, secondary testing of auditory and speech perception, and cognitive performance of the larger SICLID sample (*n* = 146) will be reported in greater detail elsewhere.

### Behavioral Tests

#### Bergen Dichotic Listening Test (BDLT)

Digitally recorded test materials were provided by the Department of Biological and Medical Psychology, University of Bergen, Norway. Test materials and general procedures are described in detail elsewhere (Hugdahl et al., 2009). Listeners were seated in a sound treated booth and instructed to attend to and verbally repeat speech sounds presented via Sennheiser HD 25-1 headphones connected to a laptop PC. Control software was Direct RT. Two different CV syllables from a list of six (/ba/,/da/,/ga/,/ka/,/pa/,/ta/) were presented simultaneously, one to each ear at an initial level of 65 dB SPL. Each trial was started manually by the tester when the participant was ready. In an NF condition, the listener was asked to report the syllable they “heard best.” Alternately, the listener was asked to report only that syllable presented to the left (FL) or to the right (FR) ear.

A test session started with 12 practice trials (NF). For the first six of these (ILD = 0 dB), the listener had to repeat one of two identical syllables correctly in five/six trials to proceed. For the second six trials, different syllables were presented to each ear, ILD varied between + 15 (right louder) through -15 to 0 dB each two trials, and the listener had again to get five/six trials correct to proceed. The practice trials were repeated if a listener did not achieve the prescribed correct response rate. Five children (three LiD, two TD, in addition to the 74) were excused from the experiment when they failed to achieve the prescribed correct response rate. Data collection sessions (×6) each consisted of 36 trials containing every possible pair combination. The first two, NF sessions had 12 trials each of ILD = 0, + 15, −15. In randomized order, there followed two FR and two FL sessions, with 12 trials each of ILD = 0, −15, + 15 dB (first session), and ILD = 0, + 15, −15 dB (second session). A short break was provided between each session. Data were downloaded to REDCap (Harris et al., 2009, 2019) for storage and analysis.

#### LiSN-S

The LiSN-S task^1^ (Cameron and Dillon, 2007) measures ability to attend, hear, and recall sentences in the presence of distracting sentences. LiSN-S was administered using a laptop, a task-specific soundcard, and Sennheiser HD 215 headphones. Participants were asked to repeat a series of target sentences (“T”), presented directly in front (0°), while ignoring two distracting talkers. There were four listening conditions, in which the distractors change voice (different or same as target) and/or (virtual) position (0° and 90° relative to the listener). The test was adaptive; the level of the target speaker decreased or increased in SNR relative to the distracting talkers as the listener responded correctly or incorrectly. Testing continued for a minimum of 22 trials per condition (including five practice items that did not contribute to the score). Testing stopped when SEM < 1 or after 30 trials. The 50% correct SNR was either the “Low cue SRT” (same voice, 0° relative to the listener) or the “High cue SRT” (different voice, 90° relative to the listener). Three “derived scores” were the Talker Advantage, Spatial Advantage, and Total Advantage, so-called because each is the difference between SRTs from two conditions.

#### NIH Cognition Toolbox

Cognition was assessed using the NIH Toolbox – Cognition Domain battery of tests (Weintraub et al., 2013). Participants completed testing online or via iPad app in accordance with the current NIH recommendations in a private sound attenuated booth or quiet room. The battery contains up to seven different standardized cognitive instruments measuring different aspects of vocabulary, memory, attention, executive functioning, etc. The precise composition of the testing battery is dependent on participant age. Sixty five participants in this study completed the picture vocabulary test (TPVT), flanker inhibitory control and attention task (Flanker), DCCS test, and PSMT. Each test produced an age-corrected standardized score and the scores of all four tests were combined to calculate a single, Early Childhood Composite. Additional tests, contributing to the Crystallized, Fluid and Total Composite scores, were the LSWM, the PCPS, and the RR.

Toolbox picture vocabulary test is an adaptive test in which the participant is presented with an audio recording of a word and selects which of four pictures most closely matches the meaning of the word. In the Flanker, testing inhibition/attention, the participant reports over 40 trials the direction of a central visual stimulus (left or right, fish or arrow) in a string of five similar, flanking stimuli that may be congruent (same direction as target) or incongruent (opposite direction). The DCCS tests cognitive flexibility (switching attention). Target and test “card” stimuli vary along two dimensions, shape and color. Participants are asked to match test cards to the target card according to a specified dimension that varies for each trial. Both the Flanker and DCCS score accuracy and reaction time. PSMT assesses episodic memory by presenting an increasing number of illustrated objects and activities, each with a corresponding audio-recorded descriptive phrase. Picture sequences vary in length from 6 to 18 pictures depending on age, and participants are scored on the cumulative number of adjacent pairs remembered correctly over two learning trials.

### Magnetic Resonance Imaging

#### Stimuli and Task

fMRI scanning included an active speech categorization task. Sixteen BKB sentences (Bench et al., 1979) recorded by a single male North American speaker under studio recording conditions were presented using sparse scanning procedures (“HUSH”; Schmithorst and Holland, 2004; Deshpande et al., 2016). Specifically, sentences were presented during a 6 s silent interval followed by 6 s of fMRI scanning (details below). Following methods described by Scott et al. (2000), recordings limited to < 3.8 kHz but otherwise unprocessed were delivered as “Clear” speech sentences. “Rotated” speech stimuli were created by rotating each sentence spectrally around 2 kHz using the (Blesser, 1972) technique. Rotated speech was not intelligible, though some phonetic features and some of the original intonation were preserved. “Rotated and Vocoded” speech stimuli were created by applying six-band noise-vocoding (Shannon et al., 1995) to the rotated speech stimuli. While the rotated noise-vocoded speech was completely unintelligible, the character of the envelope and some spectral detail was preserved. The listener’s task was to make a button press after each sentence presentation, indicating whether a cartoon image (“human” or “alien”) matched the speaker of the sentence. In familiarization trials, before scanning, the clear speech was introduced as “human” and the rotated/vocoded speech as “alien.” Each participant completed three practice trials with verbal feedback from the tester. If a trial was completed incorrectly, the stimuli and instructions were reintroduced until the listener showed understanding.

#### Procedure

All listeners wore foam ear plugs to attenuate the scanner noise, but they were still able to hear clearly the stimuli delivered binaurally (diotically) via MR-compatible circumaural headphones. Listeners completed 48 matching trials, 16 of each sentence type, with no feedback. To maintain scanner timings, the sentence task continued regardless of whether a response was made. However, if a response was not made on three trials in a row, the tester provided reminders/encouragement over the scanner intercom between stimuli presentations.

#### Imaging

MRI was performed using a 3T Phillips Ingenia scanner with a 64-channel head coil and Avotec audiovisual system. The scanning protocol included a T1-weighted anatomical scan (1 mm isotropic resolution) and the fMRI task described above using a sparse acquisition approach (“HUSH”; TE = 30 ms, TR = 2000 ms, voxel size = 2.5 × 2.5 × 3.5 mm, 39 slices ascending).

### Analysis

#### Behavioral Analysis

ECLiPS, LiSN-S, and NIH Toolbox data were separately analyzed in two-way mixed effects ANOVA, with the Group variable (TD/LiD) and within-subject variables for subtests. Separate *t*-tests were used to examine composite scores.

Dichotic listening data were first analyzed in a four-way mixed effects ANOVA, with the variables 2 Groups (TD/LiD) × 2 Ear (Right, Left) × 3 Attention (NF, FR, FL) × 3 ILD (0, + 15, −15), and number of correct reports as the dependent variable. The Group variable was treated as a between-group variable, while the ear, intensity, and attention variables were treated as within-subject variables. In a second three-way ANOVA, we reduced the design to the variables Group × Attention × Intensity, and with the LI score as dependent variable. The LI score controlled for differences in overall performance between the participants, and was calculated according to the formula [(REar – LEar)/(REar + LEar) × 100].

To elucidate differences between groups in sensitivity to manipulating the physical acoustic environment of the stimuli, a third, two-way ANOVA further reduced the variables to Group × ILD, again based on the LI scores. Follow-up *post hoc* tests of main- and interaction-effects were done with Fisher’s LSD test. Significance threshold was set at *p* = 0.05 for all tests.

Correlations between DL, care-giver report, spatialized listening, and cognitive function were conducted using Pearson’s coefficient between age-corrected DL-LI across ILD, and ECLiPS Total Score, LiSN-S Low Cue and Talker Advantage, and NIH Toolbox Total Composite.

#### Imaging Analysis

First-level fMRI data were processed using FSL (FMRIB Software Library^2^). Anatomical T1 data and functional data were first reoriented using FSL’s *fslreorient2std*. Next, the T1 data were brain extracted using FSL’s BET. The brain extracted T1 image was then normalized and resampled to the 2 mm isotropic MNI ICBM 152 non-linear sixth generation template using FSL’s FLIRT. For the functional data, the initial three time points were discarded to allow protons to reach T1 relaxation equilibrium. Slice timing correction was carried out using FSL’s “*slicetimer*” and BET, respectively. Outlying functional volumes were detected using FSL’s “*fsl_motion_outliers*” with the default RMS intensity difference. Cardiac and respiration signals were regressed out using AFNI’s “*3dretroicor.*” Motion correction of the BOLD time-series was carried out using MCFLIRT. Motion-related artifacts were regressed from the data by setting up a general linear model (GLM) using six motion parameters. The amount of motion during the scans did not differ between groups.

Second level analysis was also conducted using FSL. A GLM approach was used to create group activation maps based on contrasts between conditions for all participants (i.e., regardless of LiD/TD status). Group composite images were thresholded using a family-wise error correction (*p* < 0.001) and clustering threshold of *k* = 4 voxels. Three BOLD activation contrasts were used as localizers responding to different aspects of speech perception (Halai et al., 2015 modified from Scott et al., 2000). First, the “Speech” activation map contrasts a signal having intelligibility, intonation, phonetics, and sound with one lacking all these attributes except sound (clear > rotated/vocoded). Second, the “Intelligibility” activation map contrasts a signal having all attributes with one retaining intonation, phonetics, and sound (clear > rotated). Third, the “Phonetics” activation map contrasts a signal having intonation, phonetics, and sound with one having only sound (rotated > rotated + vocoded).

#### Regions of Interest (ROI) Analysis

These three activation maps were used to identify brain regions showing significantly increased activation for speech, phonetic features, and intelligibility. These active regions were used as ROIs for correlation analysis with the DL behavioral data within which significant group differences between TD and LiD were hypothesized. Statistical analysis used JASP (v. 0.10.2) to plot data regressions and calculate correlations. Differences between correlation coefficients of each group were tested using Fisher’s r-to-z transformation.

## Results

### Audiometry and Caregiver Report

No significant difference in pure tone auditory threshold detection was found between children who were TD and those who had LiD across either the standard (Figure 1A) or extended (Figure 1B) frequency range. Children formed a continuum of listening abilities, as assessed by caregivers, but two groups, TD and LiD were segregated, primarily on their total score on the ECLiPS (Figure 1C). Two children in the LiD group who overlapped with the TD range of scores, and an additional 11 children with LiD had a clinical diagnosis of APD.

### Dichotic Listening

Children of all ages were generally able to complete the full 216 trials of BDLT testing in about 30 min, although there was a significant attrition rate as testing continued since the task is not the most engaging and fatigue was commonplace in both groups. Participants with LiD were more likely to become frustrated or upset by the task. Frequent check-ins with the participant were needed and short breaks (a few minutes) were not uncommon. However, neither fatigue nor inattention was a basis for exclusion. Forced conditions were counterbalanced.

**FIGURE 2:**
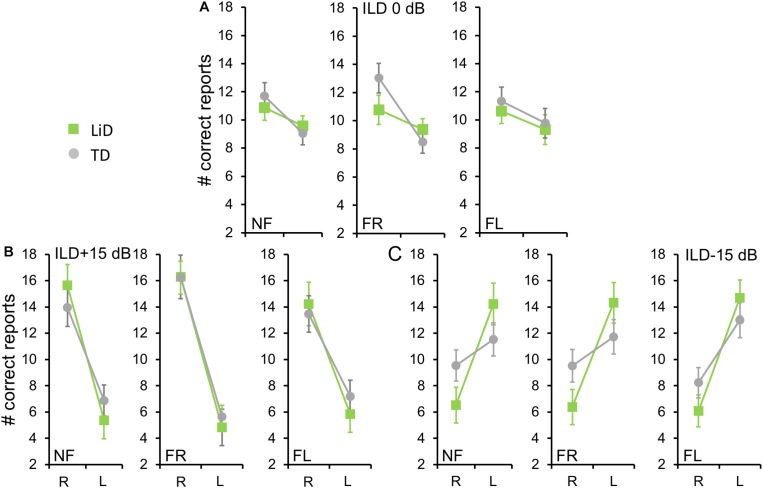
Children in both groups showed a right ear advantage in BDLT for correct syllable identification. Mean (95%CI) number of correctly identified digits delivered to each ear as a function of attention condition (NF, FL, FR), group (LiD, TD), ILD, and ear (L, R). **(A)** ILD 0 dB. Same level of stimulus in each ear. **(B)** ILD + 15 dB. Stimulus 15 dB more intense in right ear. **(C)** ILD -15 dB. Stimulus 15 dB more intense in left ear.

We first examined the BDLT results of all children in terms of number of syllables correctly identified, with a maximum possible score under each condition of 24 (12 trials × 2 blocks. 3 ILDs × 3 Attention conditions; Figure 2). For no ILD (ILD 0 dB), all three attention conditions (NF, FL, FR) showed a significant REA in both groups (Figure 2A) but there was no significant difference between attention condition. That REA became larger for ILD + 15 dB (Figure 2B) and reversed for ILD -15 dB (Figure 2C), all as expected from the DL literature, except that a REA was obtained even in the FL condition at ILD 0 dB. For the ILD -15 dB condition, it appeared that the ear differences were smaller for the TD than for the LiD group. An overall four-way ANOVA was first run with the factor “Age” as covariate to control for the small, non-significant age difference between the groups (see below). This analysis showed a significant three-way interaction of ILD by ear by group: *F*(2,142) = 5.70, *p* = 0.004, partial eta^2^ = 0.07. The interaction was followed-up with Tukey’s HSD test which showed that, while both groups were able to shift to a significant left ear advantage during the ILD -15 dB condition, this ability was exaggerated in the LiD group, controlling for multiple comparisons.

**FIGURE 3:**
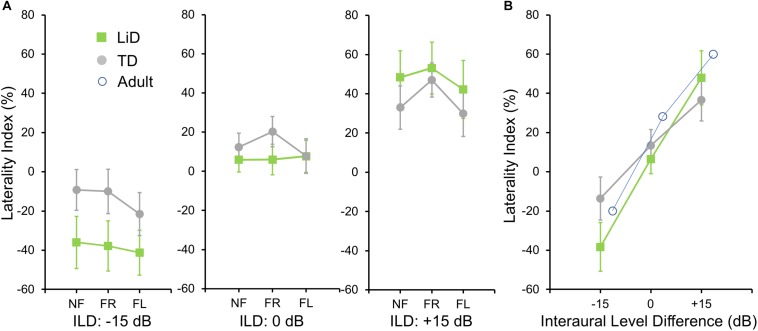
BDLT laterality index varied more in LiD group than in TD group as a function of ILD. **(A)** Same comparisons as in Figure 2 expressed as mean (95% CI) percentage correct responses right ear relative to left ear (see text). **(B)** Mean (± 95% CI) LI as a function of ILD averaged across attention conditions in each group. Adult data from Westerhausen et al. (2009).

**FIGURE 4:**
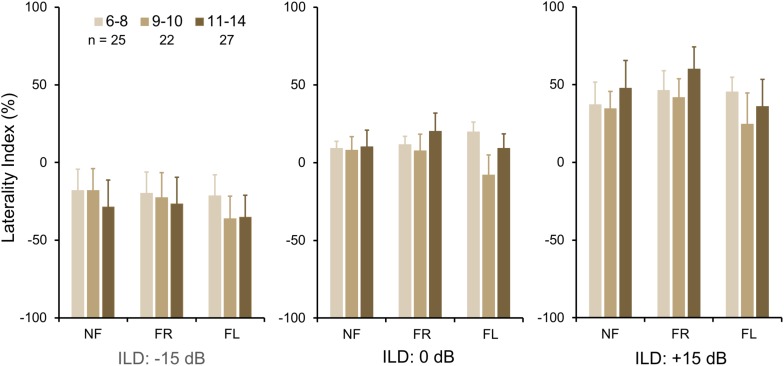
BDLT laterality index increased slightly, but non-significantly with age. Same comparisons as in Figures 2, 3; mean (95% CI) LI averaged across groups. Note that REA did not increase significantly across this age range.

To investigate group differences further, we next examined the LI (Figure 3A). Three-way ANOVA showed a significant effect of ILD: *F*(2,142) = 4.45, *p* = 0.013, partial eta^2^ = 0.06. There was a significant two-way interaction of ILD by group (Figure 3B): *F*(2,142) = 6.87, *p* = 0.001, partial eta^2^ = 0.08. Tukey’s HSD test showed significantly higher Laterality in the LiD group in the ILD −15 dB condition, controlling for multiple comparisons. Also shown on Figure 3B are typical young adult data (NF condition) from the study of Westerhausen et al. (2009). At ILD 0 dB, the LI (REA) was smallest for the LiD group (6%), larger for the TD group (13%), and largest for the Adults (28%). Note that Westerhausen’s adult data were near parallel with the LiD data, but that the LiD data showed a stronger left ear influence at each ILD. Asymmetry of LI between the ILD ± 15 dB was more marked for the TD than the LiD group, with TD children, like adults, showing a much larger LI for ILDs favoring the right ear. By contrast, children with LiD had larger but near symmetric LIs for ILD ± 15 dB. Both groups of children showed different immature response patterns. Of the 35 children with LiD who completed DL testing, 22 had been evaluated for, and nine had a diagnosis of APD. None of the means of their DL scores (reports and LI) differed significantly from that of the 26 children in the LiD group without an APD diagnosis (27 independent samples *t*-tests; *p* = 0.27–0.99). LIs for three age groups, with both TD and LiD children together and divided approximately to equalize the number of children in each group, are shown in Figure 4. As above, small differences were seen between the age groups, with older children overall having slightly larger unsigned LIs than younger children, although not significant, as indicated from three-way ANOVA with age × attention × ILD as variables. Note the positive LIs in the ILD 0 dB FL condition.

In summary, neither group, age, nor attention condition affected the LI of right/left recall. However, a significant interaction was found between group (LiD, TD) and ILD. Children with LiD were more influenced by large ILDs, especially favoring the left ear, than were TD children and were thus less able to modulate performance through attention, and more driven by the physical properties of the acoustic stimuli.

### Auditory Perceptual and Cognitive Function

**FIGURE 5:**
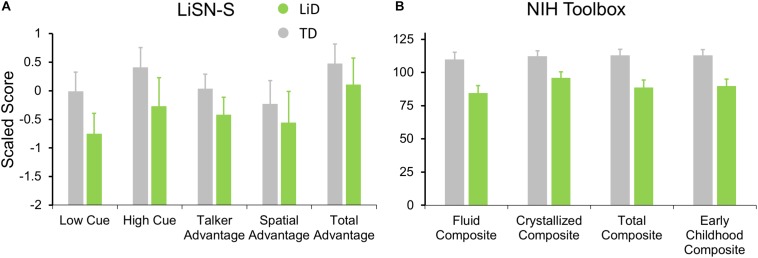
Children in LiD group had reduced speech in noise (LiSN-S) and cognitive (NIH Toolbox) performance relative to TD children. **(A)** LiSN-S (Cameron and Dillon, 2007) mean *z*-scores (±95% CI) for each group. Low Cue (speech and distractors same direction, same talker), High Cue (speech and distractors different direction, different talker), Talker advantage (benefit re Low Cue of changing distracting talker), Spatial advantage (benefit re Low Cue of changing distractor position). Further details in text. **(B)** NIH Toolbox. Mean composite scores (normalized to mean = 100, SD = 15) for each group. Further details in text.

Listening to sentences in “spatialized” noise (LiSN-S) was significantly (*p* < 0.01) impaired in children with LiD on the Low Cue and High Cue conditions, and the derived Talker Advantage measure (Figure 5A). This pattern of results suggested that the children with LiD had problems with both the procedural demands and the specifically auditory demands of the task.

Related to the disability of children with LiD to perform the listening task (LiSN-S), we found they also had impaired performance on all subtests of the NIH Toolbox, summarized in Figure 5B (all *p* < 0.001). The mean standard score of the children with LiD was poorest on the Fluid Composite, composed of the visually based NIH Toolbox DCCS, Flanker, PSM, LSWM, and PCPS subtests. Performance was also significantly impaired on the Crystallized Composite, consisting of the PV and RR subtests. The PV was the only subtest on which success was partly dependent on auditory perception and receptive language function. However, results for the PV subtest (mean difference between LiD and TD groups = 15.9 points) were similar to those of the RR subtest (16.0 points). It therefore appears that the LiD group had a generalized, multi-modal mild cognitive impairment relative to the TD group.

### Correlations Between Behavioral Measures

**FIGURE 6:**
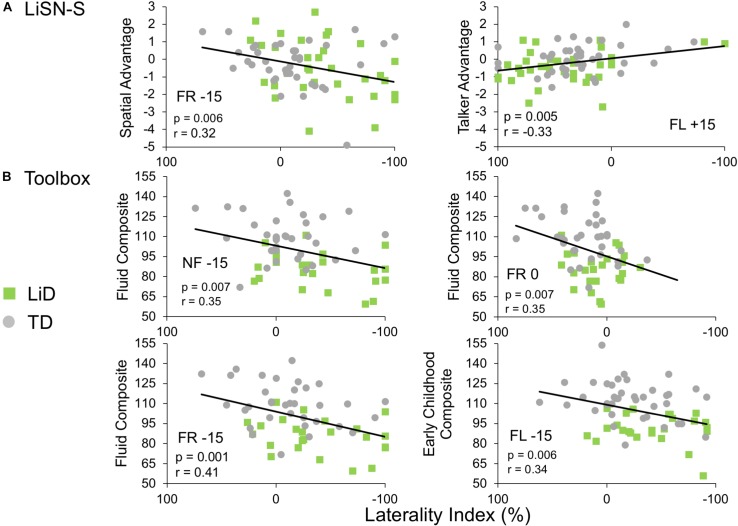
Limited correlations were seen between LI and behavioral hearing and cognitive tests. Comparative performance of individuals in both groups on **(A)** Lisn-S Advantage measures and **(B)** NIH Toolbox composite measures. Note that these were six of only nine comparisons between LI and other behavioral measures (from a total 99) that reached significance (see text).

Few significant correlations were observed between the ECLiPS, the LiSN-S or the NIH Toolbox data, and DL-LI measures. From a total of 99 comparisons, only nine LiSN-S and Toolbox measures were significant at *p* < 0.01, uncorrected for multiple comparisons. For the ECLiPS (not shown), only 3/9 comparisons were significant at *p* < 0.05, and all three comparisons were for ILD −15 dB, at which LIs and differences between groups were largest (Figure 3B). Similar patterns were seen for the LiSN-S and the Toolbox (Figure 6). Correlations between LiSN-S Spatial and Talker Advantage with LI during FR −15 and FL + 15 were just significant, after Bonferroni adjustment (two of 18 comparisons, *p* ≤ 0.006). Toolbox Composite data showed some strong and consistent correlations (Figure 6B). For example, the Fluid Composite was significantly associated with LI (*p* < 0.001) for FR −15; all four Toolbox measures showed low cognitive performance associated with strongly negative LI.

### fMRI

**FIGURE 7:**
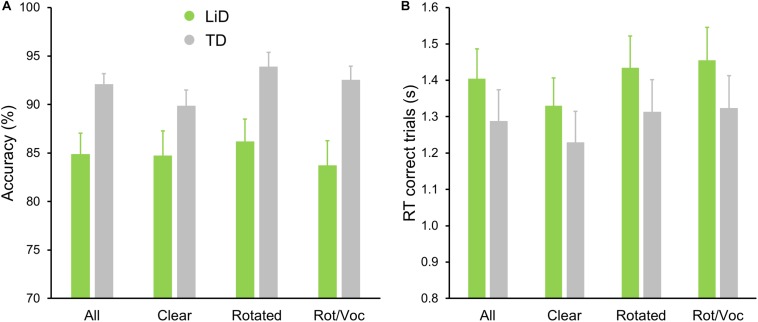
TD group showed superior performance on MRI real/unreal speech discrimination. **(A)** Mean (±SEM) percent accuracy across sentences and **(B)** reaction times (RT, in seconds) for correct trials.

All children performed well in the scanner on the active speech categorization task, although the TD children performed more accurately, and with shorter reaction times, than those with LiD (Figure 7).

**FIGURE 8:**
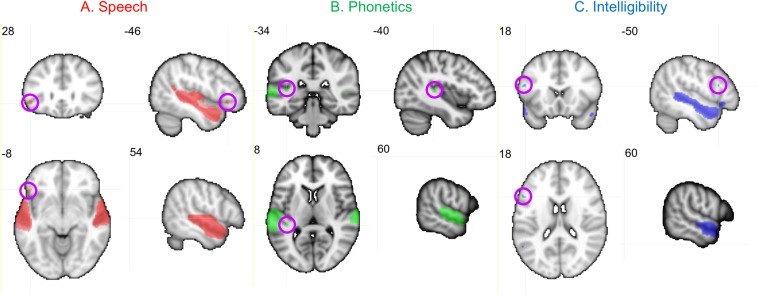
Activation produced by Speech, Phonetic, and Intelligibility processing during the speech categorization task. Family-wise error correction of *p* < 0.001 and clustering threshold of *k* = 4 voxels was applied to create key ROIs for correlations with DL behavioral outcomes. Images are in neurological orientation and slice values refer to MNI coordinates.

Neural activity associated with listening to the Speech, Phonetic, and Intelligibility of the sentences did not significantly differ between the two groups. BOLD activation from across all children (regardless of group) is shown in Figure 8.

**FIGURE 9:**
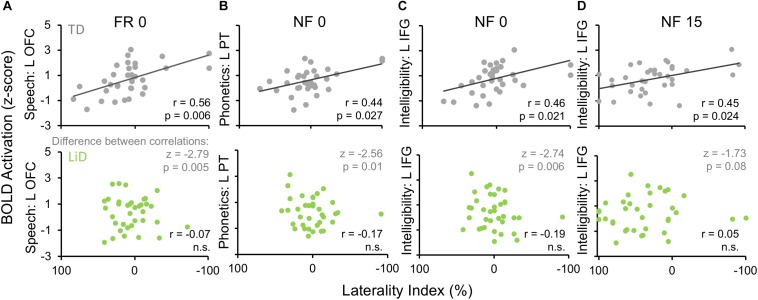
Selected, significant correlations between BDLT LI and brain activation in ROIs (Figure 8), focusing on significant activation in TDs and equivalent ROI non-activations in LiD. *r* = correlation coefficients, *z* = difference between group correlation coefficients for each region. All *p*-values in this figure have been adjusted to compensate for multiple comparisons. Further details in text.

Activation patterns for Speech included bilateral auditory cortices (middle temporal gyrus, superior temporal gyrus, and Heschl’s gyrus), PT, left temporal fusiform cortex, inferior temporal gyrus, OFC, and right parahippocampal gyrus. In the left OFC, a significant correlation (*r*) was observed among the TD children between BOLD activation and the BDLT FR0 attention condition (Figure 9A). In contrast, children with LiD lacked such a correlation.

Phonetic activation was seen in a more restricted region of posterior AC in the STG and PT bilaterally and right Heschl’s gyrus (Figure 8B). Left PT activation was correlated with the BDLT LI in TD children for the NF0 attention condition (Figure 9B). For children with LiD, the relationship flipped, but not to the extent of a significant correlation.

The Intelligibility contrast revealed increased activation in the superior temporal gyrus, with a long anterior to posterior profile from the left temporal pole and along the STG and a more anterior temporal pole locus on the right (Figure 8C). Significant correlations with BDLT LI were seen with the left IFG under both NF0 and NF15 conditions in the TD children. Again, no such correlations were observed in the children with LiD (Figures 9C,D).

The difference between the two groups in correlations of brain activation with attention conditions was significant (z) for speech-FR0 (*p* = 0.005), phonetics-NF0 (*p* = 0.01), and intelligibility-NF0 (*p* = 0.006) but not intelligibility-NF15.

## Discussion and Conclusion

### Listening Difficulties

Children with LiD performed normally in the BDLT Listen (NF) mode, as hypothesized, but they also performed normally in the BDLT Concentrate (FL, FR) modes, despite significantly impaired performance on speech-in-noise and cognitive tests. This is a new finding since most previous research on auditory processing differences between TD and non-TD children has focused on specific impairments in processing capacity of the left hemisphere, reflected in differential scores in the FL mode (Westerhausen and Hugdahl, 2010). The normal performance of children with LiD in the ILD 0 dB condition suggests that their cognitive insufficiency did not prevent them performing the DL task. Moreover, no significant differences were found between the groups on the right ear or left ear scores, suggesting no systematic hemispheric processing differences. Rather, the children with LiD were found to have a generalized disability to benefit from ILDs between the dichotic stimuli. The small REA found in both groups is consistent with weak REAs reported in a previous study of young children (Passow et al., 2013).

Performance on BDLT of children with LiD was more affected by varying ILD than was that of TD children. This could be because the children with LiD had a primary auditory problem, or that they were less able to offset greater sound level at either ear through attention modulation (Westerhausen and Hugdahl, 2010). Poor LiSN-S performance, particularly on the spatial advantage measure, may indicate a binaural interaction problem (Cameron and Dillon, 2008; Glyde et al., 2013; Cameron et al., 2014). Correlations between LI and LiSN-S advantage measures at high ILD support this interpretation, but LiD and TD groups did not differ in this respect. Inattention is a primary symptom of LiD, although its relationship to APD is controversial (Moore et al., 2010; Moore, 2018). However, there seems to be general agreement that many if not most children undergoing APD evaluation have attention difficulties that, at least, need to be taken into account by the examining audiologist (American Academy of Audiology, 2010; Sharma et al., 2014; Moore et al., 2018).

### Age

There have been few studies of children using the BDLT. Age effects in this study generally mirrored those previously reported (Hugdahl et al., 2001; Passow et al., 2013), although the REA tended to get larger with increasing age, in contrast to a recent, NF-only study (Kelley and Littenberg, 2019). In fact, comparison between current LiD data and adult data of Westerhausen et al. (2009) suggested a robust, consistent increase in right ear influence with age, across ILD, supporting a “right ear weakness” hypothesis for LiD. This contrasted with TD children who had more of a “REA amplification” pattern of development where changes in LI with increasing age were asymmetric between left- and right-leading ILDs.

### fMRI

Both groups of children (LiD, TD) used the same brain areas to perform the sentence-listening task but relations between brain area activation and BDLT LI suggested the areas are used differently by the two groups. Left OFC was related to LI forced attention for TD but not for LiD. Other areas (left PT and IFG) also related to LI activation in TD, but not LiD. These results were all somewhat independent of ILD or task type (NF or forced). As outlined in the Introduction, it was predicted that, if the LiD children have an auditory processing deficit, we would find similar relationships between cortical activity and behavioral results for the BDLT attention conditions (i.e., FL and FR) in both groups, and different relationships between groups for the intensity manipulations in the BDLT (i.e., −15, 0, and 15). The reverse would suggest the LiD children have language processing deficits.

A lack of group differences in BOLD activations for the sentence-listening task in the MRI scanner suggests that the groups of children do not differ in the brain areas used to process auditorily presented sentences. However, the relationships between these brain areas and BDLT laterality suggest that these brain areas are used differently by each group. In the TD group, activation in a specific cortical area used for top-down processing of speech (left OFC) was related to degree of laterality on a BDLT forced attention condition. However, this relationship was not found in the LiD group. Similarly, activation in areas used for bottom-up integration (sound, phonetics, and intonation in the left PT and IFG) was related to laterality of NF attention at different intensity levels in the TD group, but not in the LiD group. Lesser relationships were observed in other areas, with an overall pattern of some limited correlations with laterality among the TD group and lack of correlation in the LiD group.

Different group relationships were found between cortical activity and BDLT behavioral results for the attention conditions and also in the intensity manipulations. This suggests that the LiD children do not have a clear pattern of cortical reorganization associated with auditory processing. These results do not indicate a redistribution of cortical listening areas in children with LiD but, instead, a reorganization as to how these areas are engaged during language listening. Specifically, TD children showed a pattern of higher engagement of specific cortical listening areas used to support better listening task performance. This pattern was not observed in children with LiD.

### Implications for Listening Difficulties in Children

Clinical use of dichotic assessment for APD in children has mostly used dichotic digits (Musiek, 1983; Emanuel et al., 2011). As discussed in Section “Introduction,” the results of that assessment do not distinguish between an auditory and a cognitive explanation of those children’s LiD, although the test may correctly identify children without hearing loss as having an auditory perceptual or speech coding problem. In other studies, using the less cognitively but more auditorily demanding BDLT, older children and adults with a wide variety of learning, neurological, and mental health diagnoses had a generally weak left ear performance in the FL condition (Westerhausen and Hugdahl, 2010). This was interpreted as a means for testing top-down executive function that we found here, in the Toolbox data, to be impaired in children with LiD. However, we did not find a consistent poor performance on the FL task in that group.

A number of observations have been made about dichotic ear advantages in children with APD (Moncrieff et al., 2017). Some studies have focused on the prevalence of a REA or LEA, suggesting balance between the two is initially more even but unstable, but that a consistent REA emerges with age (Moncrieff, 2011). However, the current results, and others (Hugdahl et al., 2001) show that the absolute level of LI increases (i.e., larger LEA and REA) with age, contrary to the report of Moncrieff (2011), and that use of a binary LEA/REA distinction can be misleading. Other results in adults have shown that larger LIs, either positive or negative, are associated with better accuracy on the BLDT (Hirnstein et al., 2014).

A new term, “amblyaudia” was introduced by Moncrieff et al. (2016) to designate “an abnormally large asymmetry between the two ears during DL tasks with either normal or below normal performance in the dominant ear.” The results of the study reported here only partially supported amblyaudia in the children with LiD. Their performance was statistically indistinguishable from that of the TD children at ILD 0 dB, the usual condition for testing. But, consistent with the definition, there was a larger than normal asymmetry in the ILD 15 dB conditions, with normal or below normal number of correctly reported digits in the right ear and a greater than normal number of correct reports in the left ear. In a review, Whitton and Polley (2011) discussed amblyaudia in the context of long-term effects of conductive hearing loss on auditory system plasticity, induced in children predominantly by otitis media. While building a convincing case from the literature for such plasticity, the relevance of such findings to the children in this study is unclear; a similar proportion of children in each group had a history of PE tubes (Hunter et al., 2020).

Several forms of dichotic training have been proposed, and we know of at least two that are in current evaluation or practice for the treatment of amblyaudia (DIID—Musiek, 2004; ARIA—Moncrieff et al., 2017) and other abnormalities detected through DL evaluation (Emanuel et al., 2011). Unfortunately, it remains unclear what sort of benefits might be obtained from such training or whether any of the proposed methods generalize to improved listening in real-world challenging environments. Hugdahl et al. (2009) present several arguments *against* training using the BDLT to treat impaired performance on the FL instruction task. These arguments are, briefly, that the DL task is very simple and therefore unchallenging, that it shows little or no learning effect, and that executive functions are not amenable to training. It is unclear what form of training might be useful for normalizing specific DL behavior patterns of the children with LiD in the current study. However, interventions that improve auditory attention should be generally useful for these children.

In summary, we found little evidence for impaired DL on the BDLT or brain activation differences for children with LiD compared with TD children. A significant reduction of the LI was found in the LiD group when the left ear stimulus was presented at a reduced level compared with the right ear stimulus. Brain activation was correlated with LI in some frontal and temporal regions for children in the TD group, but not for those in the LiD group.

## Data Availability Statement

The datasets generated for this study are available on request to the corresponding author.

## Ethics Statement

The studies involving human participants were reviewed and approved by the Cincinnati Children’s Institutional Review Board (IRB). Written informed consent to participate in this study was provided by the participants’ legal guardian/next of kin.

## Author Contributions

DM, KH, LH, and HS conceived and designed the study. AP, NS, and EC performed the behavioral testing and analyzed the data. HS and JV performed the MRI and analyzed the data. All authors contributed to the writing.

## Conflict of Interest

The authors declare that the research was conducted in the absence of any commercial or financial relationships that could be construed as a potential conflict of interest.

## Footnotes

1 www.LiSN-S.com

2 https://www.fsl.fmrib.ox.ac.uk/fsl/

## Abbreviations

(C)APD: (central) auditory processing disorder
BDLT: Bergen Dichotic Listening Test
CV: consonant vowel
DCCS: Dimensional Change Card Sort Test
DL: dichotic listening
FL: forced left
FR: forced right
IFG: inferior frontal gyrus
ILD: interaural level difference IRB Institutional Review Board
LI: Laterality Index
LiD: listening difficulties
LiSN-S: Listening in Spatialized Noise
Sentences: Test
LSWM: List Sorting Working Memory Test
NF,: non-forced OFC orbitofrontal cortex
PCPS, Pattern Comparison Processing Speed Test: PSMT Picture Sequence Memory Test
PT: planum temporale
REA: right ear advantage
ROI: region of interest
RR: Oral Reading Recognition Test
SICLID: Sensitive Indicators of Childhood Listening Difficulties
SNR: signal/noise ratio
SRT: speech reception threshold
TD: typically developing
TPVT: Toolbox picture vocabulary test
y.o.: years old.

## References

[B1] American Academy of Audiology (2010). *Clinical Practice Guidelines: Diagnosis, Treatment, and Management of Children and Adults With Central Auditory Processing Disorder.* Available at: https://audiology-web.s3.amazonaws.com/migrated/CAPD%20Guidelines%208-2010.pdf_539952af956c79.73897613.pdf (accessed December16, 2019).

[B2] Barry J. G. Moore D. R. (2015). *Evaluation of Children’s Listening and Processing Skills (ECLIPS).* London: Mrc-T Publishing.

[B3] Barry J. G. Tomlin D. Moore D. R. Dillon H. (2015). Use of questionnaire-based measures in the assessment of listening difficulties in school-aged children. *Ear Hear.* 36:e00300-13. 10.1097/AUD.0000000000000180 PMC4617294 26002277

[B4] Bench J. Kowal A. Bamford J. (1979). The Bkb (Bamford-Kowal-Bench) sentence lists for partially-hearing children. *Br. J. Audiol.* 13 108–112. 10.3109/03005367909078884 486816

[B5] Blesser B. (1972). Speech perception under conditions of spectral transformation. I. Phonetic characteristics. *J. Speech Hear. Res.* 15 5–41. 10.1044/jshr.1501.05 5012812

[B6] Brenneman L. Cash E. Chermak G. D. Guenette L. Masters G. Musiek F. E. (2017). The relationship between central auditory processing, language, and cognition in children being evaluated for central auditory processing disorder. *J. Am. Acad. Audiol.* 28 758–769. 10.3766/jaaa.16119 28906246

[B7] Broadbent D. E. (1956). Successive responses to simultaneous stimuli. *Q. J. Exp. Psychol.* 8 145–152. 10.1080/17470215608416814

[B8] Cameron S. Dillon H. (2007). Development of the listening in spatialized noise-sentences test (Lisn-S). *Ear Hear.* 28 196–211. 10.1097/aud.0b013e318031267f 17496671

[B9] Cameron S. Dillon H. (2008). The listening in spatialized noise-sentences test (LISN-S): comparison to the prototype LISN and results from children with either a suspected (central) auditory processing disorder or a confirmed language disorder. *J. Am. Acad. Audiol.* 19 377–391. 10.3766/jaaa.19.5.2 19253811

[B10] Cameron S. Dillon H. (2020). Are “Dichotic” deficits uniquely dichotic? investigating dichotic performance with the dichotic digits difference test (DddT) in a large clinical population of children referred for an auditory processing assessment. *J. Am. Acad. Audiol.* 10.376/jaaa.19037 [Epub ahead of print]. 32119818

[B11] Cameron S. Dillon H. Glyde H. Kanthan S. Kania A. (2014). Prevalence and remediation of spatial processing disorder (SPD) in Indigenous children in regional Australia. *Int. J. Audiol.* 53 326–335. 10.3109/14992027.2013.871388 24471411

[B12] Cameron S. Glyde H. Dillon H. Whitfield J. Seymour J. (2016). The dichotic digits difference test (DddT): development, normative data, and test-retest reliability studies part 1. *J. Am. Acad. Audiol.* 27 458–469. 10.3766/jaaa.15084 27310404

[B13] de Wit E. Visser-Bochane M. I. Steenbergen B. Van Dijk P. Van Der Schans C. P. Luinge M. R. (2016). Characteristics of auditory processing disorders: a systematic review. *J. Speech Lang. Hear Res.* 59 384–413. 27082630 10.1044/2015_JSLHR-H-15-0118

[B14] Deshpande A. K. Tan L. Lu L. J. Altaye M. Holland S. K. (2016). fmri as a preimplant objective tool to predict postimplant oral language outcomes in children with cochlear implants. *Ear Hear.* 37 e263–e272. 10.1097/aud.0000000000000259 26689275

[B15] Emanuel D. C. Ficca K. N. Korczak P. (2011). Survey of the diagnosis and management of auditory processing disorder. *Am. J. Audiol.* 20 48–60. 10.1044/1059-0889(2011/10-0019) 21474554

[B16] Glyde H. Buchholz J. M. Dillon H. Cameron S. Hickson L. (2013). The importance of interaural time differences and level differences in spatial release from masking. *J. Acoust. Soc. Am.* 134 EL147–EL152. 10.1121/1.4812441 23927217

[B17] Halai A. D. Parkes L. M. Welbourne S. R. (2015). Dual-echo fMRI can detect activations in inferior temporal lobe during intelligible speech comprehension. *Neuroimage* 122 214–221. 10.1016/j.neuroimage.2015.05.067 26037055 PMC4627358

[B18] Harris P. A. Taylor R. Minor B. L. Elliott V. Fernandez M. O’Neal L. (2019). The Redcap consortium: Building an international community of software platform partners. *J. Biomed. Inform.* 95:103208 10.1016/j.jbi.2019.103208 PMC7254481 31078660

[B19] Harris P. A. Taylor R. Thielke R. Payne J. Gonzalez N. Conde J. G. (2009). Research electronic data capture (Redcap)–a metadata-driven methodology and workflow process for providing translational research informatics support. *J. Biomed. Inform.* 42 377–381. 10.1016/j.jbi.2008.08.010 18929686 PMC2700030

[B20] Harris V. L. Keith R. W. Novak K. K. (1983). Relationship between two dichotic listening tests and the Token test for children. *Ear Hear.* 4 278–282. 10.1097/00003446-198311000-00003 6653930

[B21] Hind S. E. Haines-Bazrafshan R. Benton C. L. Brassington W. Towle B. Moore D. R. (2011). Prevalence of clinical referrals having hearing thresholds within normal limits. *Int. J. Audiol.* 50 708–716. 10.3109/14992027.2011.582049 21714709

[B22] Hirnstein M. Hugdahl K. Hausmann M. (2014). How brain asymmetry relates to performance - a large-scale dichotic listening study. *Front. Psychol.* 4:997 10.3389/fpsyg.2013.00997 PMC3877751 24427151

[B23] Hugdahl K. Bronnick K. Kyllingsbaek S. Law I. Gade A. Paulson O. B. (1999). Brain activation during dichotic presentations of consonant-vowel and musical instrument stimuli: a 15O-Pet study. *Neuropsychologia* 37 431–440. 10.1016/s0028-3932(98)00101-8 10215090

[B24] Hugdahl K. Carlsson G. Eichele T. (2001). Age effects in dichotic listening to consonant-vowel syllables: interactions with attention. *Dev. Neuropsychol.* 20 445–457. 10.1207/s15326942dn2001_8 11827098

[B25] Hugdahl K. Westerhausen R. (2016). Speech processing asymmetry revealed by dichotic listening and functional brain imaging. *Neuropsychologia* 93 466–481. 10.1016/j.neuropsychologia.2015.12.011 26706774

[B26] Hugdahl K. Westerhausen R. Alho K. Medvedev S. Hamalainen H. (2008). The effect of stimulus intensity on the right ear advantage in dichotic listening. *Neurosci. Lett.* 431 90–94. 10.1016/j.neulet.2007.11.046 18162310

[B27] Hugdahl K. Westerhausen R. Alho K. Medvedev S. Laine M. Hamalainen H. (2009). Attention and cognitive control: unfolding the dichotic listening story. *Scand. J. Psychol.* 50 11–22. 10.1111/j.1467-9450.2008.00676.x 18705670

[B28] Hunter L. L. Blankenship C. M. Sloat N. T. Perdew A. Stewart H. J. Moore D. R. (2020). Peripheral auditory involvement in childhood listening difficulty. *Ear Hear.* (in press). 10.1097/AUD.0000000000000899 PMC7759582 32740300

[B29] Keith R. W. (2009). *Scan–3:C Tests for Auditory Processing Disorders for Children.* London: Pearson.

[B30] Keith R. W. Tektas M. Ramsay K. Delaney S. (2019). Development and standardization of the university of cincinnati auditory processing inventory (UCAPI). *Int. J. Audiol.* 58 373–378. 10.1080/14992027.2019.1585973 30939055

[B31] Kelley K. S. Littenberg B. (2019). Dichotic listening test-retest reliability in Children. *J. Speech Lang. Hear. Res.* 62 169–176. 10.1044/2018_jslhr-h-17-0158 30950751

[B32] Kimura D. (1961). Cerebral dominance and the perception of verbal stimuli. *Can. J. Psychol.* 15 166–171. 10.1037/h0083219

[B33] Kimura D. (1963). Speech lateralization in young children as determined by an auditory test. *J. Comp. Physiol. Psychol.* 56 899–902. 10.1037/h0047762 14050184

[B34] Kompus K. Specht K. Ersland L. Juvodden H. T. Van Wageningen H. Hugdahl K. (2012). A forced-attention dichotic listening fmri study on 113 subjects. *Brain Lang.* 121 240–247. 10.1016/j.bandl.2012.03.004 22494771

[B35] Moncrieff D. Keith W. Abramson M. Swann A. (2016). Diagnosis of amblyaudia in children referred for auditory processing assessment. *Int. J. Audiol.* 55 333–345. 10.3109/14992027.2015.1128003 27058650

[B36] Moncrieff D. Keith W. Abramson M. Swann A. (2017). Evidence of binaural integration benefits following Aria training for children and adolescents diagnosed with amblyaudia. *Int. J. Audiol.* 56 580–588. 10.1080/14992027.2017.1303199 28346034

[B37] Moncrieff D. W. (2011). Dichotic listening in children: age-related changes in direction and magnitude of ear advantage. *Brain Cogn.* 76 316–322. 10.1016/j.bandc.2011.03.013 21530051

[B38] Moore D. R. (2018). Editorial: auditory processing disorder. *Ear Hear.* 39 617–620. 10.1097/aud.0000000000000582 29664753 PMC6124895

[B39] Moore D. R. Ferguson M. A. Edmondson-Jones A. M. Ratib S. Riley A. (2010). Nature of auditory processing disorder in children. *Pediatrics* 126 e382–e390. 20660546 10.1542/peds.2009-2826

[B40] Moore D. R. Hunter L. L. (2013). Auditory processing disorder (APD) in children: a marker of neurodevelopmental syndrome. *Hear. Balance Commun.* 11 160–167. 10.3109/21695717.2013.821756

[B41] Moore D. R. Hutchings M. E. Meyer S. E. (1991). Binaural masking level differences in children with a history of otitis media. *Audiology* 30 91–101. 10.3109/00206099109072874 1877902

[B42] Moore D. R. Sieswerda S. L. Grainger M. M. Bowling A. Smith N. Perdew A. (2018). Referral and diagnosis of developmental auditory processing disorder in a large, United States hospital-based audiology service. *J. Am. Acad. Audiol.* 29 364–377. 10.3766/jaaa.16130 29708487

[B43] Musiek F. E. (1983). Assessment of central auditory dysfunction: the dichotic digit test revisited. *Ear Hear.* 4 79–83. 10.1097/00003446-198303000-00002 6840415

[B44] Musiek F. E. (2004). The Diid: a new treatment for Apd. *Hear. J.* 57:50 10.1097/01.hj.0000293049.80297.cd

[B45] Passow S. Muller M. Westerhausen R. Hugdahl K. Wartenburger I. Heekeren H. R. (2013). Development of attentional control of verbal auditory perception from middle to late childhood: comparisons to healthy aging. *Dev. Psychol.* 49 1982–1993. 10.1037/a0031207 23276126

[B46] Pillsbury H. C. Grose J. H. Hall J. W. III (1991). Otitis media with effusion in children. Binaural hearing before and after corrective surgery. *Arch. Otolaryngol. Head Neck Surg.* 117 718–723. 10.1001/archotol.1991.01870190030008 1863436

[B47] Rees A. Palmer A. R. (2010). *The Auditory Brain.* Oxford: Oxford University Press.

[B48] Rimol L. M. Specht K. Weis S. Savoy R. Hugdahl K. (2005). Processing of sub-syllabic speech units in the posterior temporal lobe: an fmri study. *Neuroimage* 26 1059–1067. 10.1016/j.neuroimage.2005.03.028 15894493

[B49] Roebuck H. Barry J. G. (2018). Parental perception of listening difficulties: an interaction between weaknesses in language processing and ability to sustain attention. *Sci. Rep.* 8:6985. 10.1038/s41598-018-25316-9 PMC5934397 29725027

[B50] Roeser R. J. Millay K. K. Morrow J. M. (1983). Dichotic consonant-vowel (Cv) perception in normal and learning-impaired children. *Ear Hear.* 4 293–299. 10.1097/00003446-198311000-00006 6653933

[B51] Schmithorst V. J. Holland S. K. (2004). Event-related fmri technique for auditory processing with hemodynamics unrelated to acoustic gradient noise. *Magn. Reson. Med.* 51 399–402. 10.1002/mrm.10706 14755667

[B52] Scott S. K. Blank C. C. Rosen S. Wise R. J. (2000). Identification of a pathway for intelligible speech in the left temporal lobe. *Brain* 123 (Pt 12), 2400–2406. 10.1093/brain/123.12.2400 11099443 PMC5630088

[B53] Shannon R. V. Zeng F. G. Kamath V. Wygonski J. Ekelid M. (1995). Speech recognition with primarily temporal cues. *Science* 270 303–304. 10.1126/science.270.5234.303 7569981

[B54] Sharma M. Dhamani I. Leung J. Carlile S. (2014). Attention, memory, and auditory processing in 10- to 15-year-old children with listening difficulties. *J. Speech Lang. Hear. Res.* 57 2308–2321. 10.1044/2014_jslhr-h-13-0226 25198800

[B55] Weihing J. Guenette L. Chermak G. Brown M. Ceruti J. Fitzgerald K. (2015). Characteristics of pediatric performance on a test battery commonly used in the diagnosis of central auditory processing disorder. *J. Am. Acad. Audiol.* 26 652–669. 10.3766/jaaa.14108 26218054

[B56] Weintraub S. Dikmen S. S. Heaton R. K. Tulsky D. S. Zelazo P. D. Bauer P. J. (2013). Cognition assessment using the Nih Toolbox. *Neurology* 80 S54–S64. 23479546 10.1212/WNL.0b013e3182872ded PMC3662346

[B57] Westerhausen R. Hugdahl K. (2010). “Cognitive control of auditory laterality,” in *The Two Halves of the Brain: Information Processing in the Cerebral Hemispheres*, eds Hugdahl K. Westerhausen R. (Cambridge: Mit Press).

[B58] Westerhausen R. Moosmann M. Alho K. Belsby S. O. Hamalainen H. Medvedev S. (2010). Identification of attention and cognitive control networks in a parametric auditory fmri study. *Neuropsychologia* 48 2075–2081. 10.1016/j.neuropsychologia.2010.03.028 20363236

[B59] Westerhausen R. Moosmann M. Alho K. Medvedev S. Hamalainen H. Hugdahl K. (2009). Top-down and bottom-up interaction: manipulating the dichotic listening ear advantage. *Brain Res.* 1250 183–189. 10.1016/j.brainres.2008.10.070 19028471

[B60] Whitton J. P. Polley D. B. (2011). Evaluating the perceptual and pathophysiological consequences of auditory deprivation in early postnatal life: a comparison of basic and clinical studies. *J. Assoc. Res. Otolaryngol.* 12 535–547. 10.1007/s10162-011-0271-6 21607783 PMC3173557

[B61] Wilson W. J. (2018). Evolving the concept of Apd. *Intern. J. Audiol.* 57 240–248. 10.1080/14992027.2017.1409438 29390910

